# Psychologists’ involvement in and experiences of treating patients with stress-related exhaustion in primary care

**DOI:** 10.1186/s12875-024-02287-7

**Published:** 2024-02-12

**Authors:** Susanne Ellbin, Agneta Lindegård, Ingibjörg H. Jonsdottir, Elisabeth Dahlborg

**Affiliations:** 1 The Institute of Stress Medicine, Carl Skottsbergs gata 22B, Gothenburg, SE-413 19 Sweden; 2 School of Public Health and Community Medicine, Institute of Medicine, Sahlgrenska Academy, Gothenburg University, Gothenburg, Sweden; 3 Department of Medicine, Sahlgrenska University Hospital, Gothenburg, Sweden; 4 University West, Gustava Melins gata 2, SE-461 86 Trollhättan, Sweden

**Keywords:** Burnout, Exhaustion, Equal care, Primary care, Psychologist

## Abstract

**Background:**

Primary health care is the setting for most patients with stress-related mental health problems. Good care processes are important for patients with stress-related mental health problems and the complex needs of these patients has become a challenge for primary care settings which is traditionally designed to manage acute episodes of one illness. The care process of these patients is thus interesting to investigate. The aim of this study was to explore psychologists´ involvement and experiences regarding the organisation of the care process and treatment of patients seeking care for stress-related exhaustion.

**Method:**

Fifteen psychologists (14 women and 1 man, age range 27–72 years)c from fifteen different primary health care centres in the western part of Sweden, located in both rural and urban areas were included. Qualitative content analysis of individual semi-structured interviews was conducted.

**Results:**

The analysis resulted in eight subcategories within the two main categories studied illuminating psychologists’ involvement and experiences regarding the organisation of the care process and challenges regarding treatment of patients seeking care for stress-related exhaustion.

**Conclusion:**

The care process of patients with stress-related exhaustion is perceived to be ineffective and not congruent with the needs of the patients. A lack of holistic overview of the care process, a lack of collaboration and poor utilization of the health care professionals’ competence leads to an unstructured process forcing the patients to be the carriers and coordinators of their own care.

**Supplementary Information:**

The online version contains supplementary material available at 10.1186/s12875-024-02287-7.

## Introduction

Since 2010 stress-related diagnoses have been the fastest growing cause of sick leave in Sweden according to the Swedish Social Insurance Agency [1]. The increase in sick leave due to stress at work is also a general trend seen across Europe and the societal costs are high [2]. A variety of partly overlapping concepts are used both in the literature but also in clinical settings to describe stress-related exhaustion. In Sweden, the criteria-based diagnosis of Exhaustion disorder (ED) (ICD-10 code F43.8 A) is used to define patients seeking care for stress-related exhaustion (National Board of Health and Welfare, 2003). The ED diagnosis overlaps with the burnout concept which is a psychological construct based solely on work-related stress [3]. Burnout has been questioned as a diagnostic tool in clinical practice [4] but burnout is now included in the 11th Revision of the International Classification of Diseases as an occupational phenomenon but is not classified as a medical condition (ICD-11 code QD85).

Patients with ED report vast number of somatic and mental symptoms and co-morbidity with depression and anxiety is common [5]. Multifactorial stress exposure is reported by the patients [6] and a large study mapping symptoms of more than 500 individuals with self-reported ED showed a complex picture encompassing several physical, cognitive, and emotional symptoms [7]. Thus, a thorough assessment of patients with stress-related exhaustion is important and generally assessment of patients with mental health problems has been raised as challenge for practitioners [8–10]. The characterization of these patients should involve investigation of vulnerability and protective factors, identifying stressors that can either be eliminated or must be accepted, understanding what maintains the condition, understanding the strategies used by different patients, and determining suitable treatment. This characterization as well as treatment planning and the execution of therapeutic interventions, falls within the psychologist’s domain and thus makes the psychologist particularly important for the primary health care team working with these patients. The fact that the primary health care is the setting for most patients with stress-related mental exhaustion presents a number of challenges since that primary care is traditionally designed to manage acute episodes of one illness and is thus not adapted to meet the needs of patients with such complex needs and/or multimorbidity [11]. Different studies confirm that both health care professional and managers raise the issue that fundamental need for change is required in primary care, including increased resources to meet the challenges faced in primary care context [12–14]. Good care processes are important for patients with mental health problems and the complex needs of these patients has become a challenge for primary care settings. Many authors have raised the importance of collaborative care, involving different professions, as one of many solutions in how to manage the care of patients with complex needs [15, 16]. However, the knowledge regarding how well the care process is working for these patients is poor. Of interest is to explore how treatment methods are chosen given that the evidence for effective treatment for patients with stress-related mental health problems is scarce [17, 18]. It is also important to illustrate the involvement of different professions in the care process and the interplay between different professions. Thus, a large interview study was conducted exploring the perception of different professions; physicians, psychologists, and rehabilitation centre professionals (physiotherapists and occupational therapists), regarding their involvement and experience of the care process for patients seeking care for stress-related exhaustion in primary care. Here we present the part of the interview study that focus on the experience perceived by the psychologists. As mentioned above, the role of the psychologist can be considered as particularly important for patients with stress-related mental health problems. Thus, the aim of this study was to describe the experiences of psychologists regarding the organisation of the care process and the treatment of patients seeking care for stress-related exhaustion in Primary health care centres (PHCC) in Western Sweden.

## Methods

The study has a qualitative design, realised as an interview study. The aim of the study formed the basis of a semi-structured question guide with two main subjects: organization and treatment (Supplement 1).

### The primary care context for this study

The primary care in Region Västra Götaland, the second largest of Sweden’s 21 regions, with 1.7 million inhabitants (approximately 17% of the Swedish population), includes 220 PHCC, approximately half governed by private stakeholders. One requirement to receive a license to run a tax funded PHCC in Region Västra Götaland is to have at least one specialist physician, one licensed nurse, one district nurse and at least one licenced psychologist. Since 2015 a decision was made by the Region to start implementing a collaborative care model with a new role called a care manager responsible for the monitoring and follow-up of patients with common mental disorder in close collaboration with other health professionals [19]. A coordinator function for patients on sick leave, called a rehabilitation coordinator, has also been implemented in primary health care. The rehabilitation coordinator provides support for patients during their sick leave and during the return-to-work process [20]. The use of care manager and rehabilitation coordinator functions varies between different PHCC in the region.

### Selection of PHCCs for participation

A strategic selection of PHCCs to be contacted for this study was done based on variety regarding governance (public vs. private), location (rural vs. urban), size (number of listed patients) and Care Need Index as an indicator of socioeconomic conditions and risk of poor health for different areas [21]. In Sweden, primary health care provided by a private company is usually under contract with the county council and thus the cost of private and public primary healthcare is the same.

In total 36 of the 220 PHCCs in Region Västra Götaland were approached to inquire whether professionals working with patients seeking care for stress-related exhaustion, preferably those fulfilling the criteria for ED could participate in an interview study. The ED diagnosis is recommended to be used in Sweden for this patient group [22]. These patients can also be classified to have clinical burnout, which is used in many countries, but burnout as a psychological concept has not been proven well suited to be used in clinical practice [4]. Managers from 15 PHCCs consented that the professionals could be approached for an interview and one psychologist from each centre consented to participate in the study, 14 females and one male. Among the PHCCs finally included, 7 were run within the public sector and 8 were run as private PHCCs. Four of the PHCCs were in a rural area, and 11 in urban areas (9 in a city area and 2 in a middle-sized town). The size of the PHCCs ranged between 1,800 − 13,000 registered patients.

### Participants

Inclusion criteria for participation was being employed as a psychologist/psychotherapist (in this paper referred throughout as *psychologists*) at one of the selected PHCCs with the main assignment of working with psychological treatment. Fifteen professionals (Age range 27–72 years) were interviewed (14 women and 1 man). Among these, 11 individuals were licenced psychologists and the other four were psychotherapists with different backgrounds (one psychiatric nurse, two behavioural scientists and one physiotherapist with ergonomics as a speciality). The mean age of the participants was 47 years (range 27–72 years) and the mean years of work experience within primary care was 4.3 years (range 9 months-11 years). All participants spoke Swedish and no exclusion criteria were applied. For information about the participants see Table 1.

**Table 1:** Characteristics of the informants (*N* = 15)

Individual	Age range	Professional category	Years working in Primary Care
1	50–60	Psychologist	11
2	40–50	Psychologist	4
3	30–40	Psychologist	2
4	40–50	Psychologist	3–5
5	50–60	Physiotherapist/psychotherapist	6
6	missing	Psychologist	2
7	60–70	Nurse/psychotherapist	14
8	50–60	Behavioural scientist/psychotherapist	3
9	20–30	Psychologist	3
10	30–40	Psychologist	2
11	40–50	Psychologist	11
12	30–40	Psychologist	1
13	20–30	Psychologist	1
14	40–50	Psychologist	3
15	70+	Psychologist	5

### Data collection

The interviews were conducted using a semi-structured interview guide developed by a group of experienced clinicians (psychologists, physicians, and physiotherapists) working at the Institute of Stress Medicine in Gothenburg (Supplement 1). The interview guide was used to follow the thoughts that the informant narrated, providing the opportunity to pose questions in a different order and to reformulate them to gather as much information as possible. All interviews in this study were conducted by the first author (SE), an experienced specialist psychologist with 12 years clinical experience in treating patients with ED. Each interview lasted approximately one hour, and they were conducted locally at each PHCC during the spring/summer 2019. All interviews were tape-recorded and transcribed verbatim by an experienced researcher who did not otherwise participate in the study. The interviews were coded to ensure that individual participants could not be identified. The interviews were created in Swedish and translated into English.

### Data analysis

The interviews were analysed using qualitative content analysis [23, 24], in order for the manifest as well as the latent content to become visual. The analysis encompasses four steps: (a) Meaning units in the texts are identified, (b) the meaning units are condensed and coded, (c) the codes brought together and (d) sorted into categories and sub-categories.

The analysis process started with the first author reading all the interviews to get an overall understanding of the whole content. During a second reading, it was possible to highlight meaning units corresponding to the aim of the study and the meaning units were accordingly condensed and coded. They were then assembled into categories and sub-categories based on differences and similarities. The results were validated by the other researchers by reviewing the categories and sub-categories to ensure their correspondence with the empirical data. An example of the analysis process is presented in Table 2.

**Table 2:** Examples of the analysis process

Text excerpt	Meaning unit	Code	Subcategory	Category
Individual patients … But we would have to think as a group: what do we do with that patient group?	What to do with the patient group	Manage patients	A wish for a mutual plan	Organizational prerequisites
In that case, I would say that it’s our interest. I started with mindfulness about 12 years ago… So, it has been a, initially, a passionate interest for me to engage in it. And that it has turned out to be beneficial for stress, I didn’t have that in mind back then	that it has turned out to be beneficial for stress,	Beneficial for stress	Addressing common symtoms	Psychologist´s assessment and treatment of patients with ED

QSR International’s NVivo 11 qualitative data analysis, a software program developed to help researchers to manage qualitative data [25] was used to store and analyse the data.

## Results

We explored psychologists’ perceptions of care for ED related to psychologists’ assessment and treatment of ED and organizational factors which resulted in four sub-categories for each main category (Table 3).

**Table 3:** Main categories, and sub-categories

Main Category Psychologist´s assessment and treatment of patients with ED	Main Category Organizational prerequisites
**Sub-categories**	**Sub-categories**
Deepening the medical diagnosis	Management of patients with ED depends on available professional resources
Addressing common symptoms	Lacking collaboration and communication
Encountering unique needs in treatment	A wish for a mutual plan
Right timing is difficult	The patient becomes the coordinator of their own care

### Main category 1: the psychologist´s assessment and treatment of patients with ED

The results describes how psychologists approach the task of assessing and treating patients with ED including choosing methods to work with and the timing for different interventions. Most of the informants had a positive view of their work and were satisfied with the treatment given to this patient group. However, many described the challenges linked to the complexity of the disease, where personality-related factors, traumas, and challenging external factors, including both family situations and work-related factors, often were intertwined.

#### Deepening the medical diagnosis

Diagnose is often set during the first visit to the physician, but it is not uncommon for the diagnosis to be changed after the patient has been assessed by a psychologist. In some cases, the diagnosis can also wait until after the patient´s visit to the psychologist. The psychologist´s do not have the same time constrictions as physicians, therefore they can make a more thorough assessment, often using rating scales as a complement to the clinical assessment.

*Sometimes the doctor has not considered ED but rather maybe eh, focused on that it is depression or anxiety or so. And then when I meet the patient, I think that this is clearly ED* (Participant 1).

On the other hand, once the ED diagnosis has been established, it is rarely changed.  This holds true even if, during the care process, it appears that another diagnosis would have been more appropriate, for example PTSD.

#### Addressing common symptoms

The informants used their clinical experience and their broad competence from working with different patient groups, Thus, the informants had a clear idea of components that they believed should be included in the psychological treatment to address common symptoms related to ED. They expressed similar thoughts on how to manage the patients, very much in line with the regional recommendations on the care process for ED.

The informants described how after assessment, a psychoeducation was usually performed to increase the patient’s understanding of stress reactions and associated symptoms to help the patient to structure daily activities.

*I usually emphasize the balance between activity and recovery, and the balance between sleep and rest not just sleep. And the balance between fun, unpretentious activities and demanding activities* (Participant 6).

#### Encountering unique needs in treatment

The informants emphasized that all patients are unique, describing their own individual problems as contributing factors to their exhaustion. Thus, psychotherapeutic work is unique for each patient with the aim of increasing understanding, problem solving and implementing new strategies for better functioning.

*It is extremely individual, depend on who the person is, why he/she has got sick and what the disease means for the individual* (Participant 4).

Regarding psychotherapy, most informants worked with individual psychotherapy and only a few chose manual based group therapy. According to the primary care framework, short-term psychotherapy was recommended for usually 5–10 sessions but almost all informants felt they were free to increase the number of sessions if necessary. The number of sessions varied greatly between the different PHCCs and was based either on the psychologist’s accessibility, the patient’s needs or on the structure specified by manual-based treatment.

The theoretical framework behind which treatments the informants chose to work with varied between the different PHCC and it is common to use what they are most familiar with. Thus, in some cases, psychodynamic therapy was chosen, but most informants report that they chose some form of cognitive behavioural therapy, often including mindfulness, self-compassion or Acceptance and Commitment Therapy.

*I would say that it is my interest (the method) and then it has turned out to be good for stress, and I did not have that in mind then… but that it was effective for me personally* (Participant nr 7).

Getting inspiration from colleagues or stress programmes also formed a basis for treatment and it could also happen that the informants took over a concept from a previous colleague.

*No, we have not developed anything ourselves, but instead we have inherited something, a concept which I do not think is so elaborate* (Participant nr 8).

Several of the informants expressed how the lack of evidence for treatment of ED patients was problematic. Unlike depression and anxiety with existing guidelines for treatment, psychologists lacked evidence-based guidelines for the treatment of ED.

*“I think it’s one of the least researched fields, I must say, so it’s clear that there’s a lack. I can say it like this: Oh! I would like to know that this is good, you know. It’s more like, now we have to put together what we believe is best practice in a way.”* (Participant nr 11).

#### Right timing is difficult

Most informants emphasized the importance of timing for optimal treatment effect, something they struggle with. It was crucial, for example, not to start psychotherapy before the patient had regained cognitive ability to take part in the treatment. Several informants expressed how they found it difficult to judge where the patient was in the disease process. The importance of timing was frequently discussed among the professionals.

*When do we do what? I think the timing is very important…and that it is the same for everyone* (Participant nr 14).

In cases when manual-based group treatment was used, timing was especially complicated since the time might not suit all patients. At the same time the benefits of the group process and the possibility of including more patients in treatment were raised as significant.

### Main category 2: organizational prerequisites

The result describes the management of patients with ED within primary care settings in relation to the organizational framework and limitations set by the organization. This includes involvement of different professions as well as collaboration and communication between different professions and different parts of the organization.

#### Management of patients with ED depends on available professional resources

The physician usually has the first contact with patients who seek care for symptoms of stress-related exhaustion and if needed, they internally refer the patients to other professions. However, the reason behind the referral from the physician was not always clear. Few PHCCs that participated in the study had an overall plan for the care process of ED patients, and the informants had no clear idea of which patients nor the proportion of ED patients seeking care the physicians choose to refer to the psychologist.

*No. I don’t think we get everyone. I believe we probably get a rather small portion because, and it’s really not something I know, but the feeling is that some referrals we receive are for patients who may have already been on sick leave for 1–1 ½ years by the time we find out they exist.”* (Participant nr 2).

Many informants also stated that it was unclear what governs which professional category the physicians choose to refer the patients to or when in the course of the disease this referral takes place.

*I do not know of a clear plan, but it is probably the doctor’s assessment that determines where the patients are sent… I have a hard time getting an overview, and I do not know how others do and it is not my responsibility either* (Participant nr 13).

According to the informants it was common for the physician to apply concomitant referral to both the rehabilitation centre and to the psychologist, or alternatively first to the rehabilitation centre, to see how far one can progress with that intervention.

This means that it is sometimes a matter of chance to whom the patients are referred to. Since many psychologists have a waiting list, the informants believed that patients were instead referred either to the rehabilitation centre, care managers and/or rehabilitation coordinator to speed up the process for the patient. Care managers and rehabilitation coordinators are two relatively new professional categories that are part of the professional team at the care centre.

*Primary care services have a shortage of resources, and many patients are seeking care. The system is built up with the expectancy that patients will get well faster than most of the patients actually do* (Participant nr 10).

During the interviews, it appeared that two of the PHCCs had special ways of managing patients with ED. Thus, in one of these cases the care process took place within the framework of a research-funded project and in the other case the care process of ED patients was linked to a joint education for the professionals. In these cases, a plan for step-by-step care and collaboration was made for the patients.

#### Lacking collaboration and communication

Many of the informants brought up communication and collaboration/coordination issues more broadly than just being an issue for this patient group. Thus, communication was raised as a prerequisite for good collaboration between different health care professionals, but this takes place to varying degrees at different levels within the PHCCs.

Most PHCCs do not have a structured system with regular meetings to discuss and coordinate the patients care process. At some PHCCs psychologists are situated in a specific part of the centre, and thus work relatively isolated with poor insight into the work of others, both within the psychologist group and among other professionals. For instance, several informants have worked out their own treatment concept with components relevant for this patient group, without having discussed or elaborated on the matter with other professionals.

*I have a method, but what may not be so clever is that I keep it for myself. It is not something I have shared with others in any way, which is something you could actually do…* (Participant nr 14).

However, at some PHCCs, psychosocial teams had regular meetings where referrals are discussed, and patients were assigned to different professionals and/or treatment. Physicians seldom attend these meetings, except at PHCCs where there is a connection with external project financing or joint training efforts. The care managers and the rehabilitation coordinator are sometimes involved in the care process but several of the informants expressed that the care manager function was still diffuse and had not yet found its place in the organization. On the other hand, the rehabilitation coordinator role was emphasized as a key function in that it would initiate contact with the employer early in the rehabilitation process. This made the psychologist concentrate on the therapeutic work with the patient.

*“Well, I mean, this teamwork, I don’t really think we have it for these patients. Doctor, physiotherapist, psychologist, rehab coordinator in close collaboration, that would have been very good”* (Participant 3).

Most PHCCs have access to a rehabilitation centre with physiotherapists and occupational therapists. Patients can either apply themselves to meet professionals at a rehabilitation centre or be referred by, say, a physician or a psychologist at the PHCC. The fact that rehabilitation centre and the PHCC are separate units mean that they do not have access to each other’s medical records. The informants had relatively poor insight into various treatments given by the rehabilitation centre.

*I do not know exactly who works with what there ….* (Participant nr 4)

Communication regarding the care process of a patient usually took place by reading each other’s medical records, sending internal messages, or knocking on each other’s doors. For most informants this worked well, but the collaboration varied between different individuals. For instance, some physicians were more interested in this patient category and/or more interested in collaborating with other professionals.

*We do not have team meetings, but we make sure that we meet from time to time, if the lamp outside the office does not signal occupied, we go in and just talk to them* (Participant nr 7).

Other problems with collaboration raised were related to the possibilities for referral in the cases of more serious illnesses or comorbidity that required specialist care. Some PHCCs referred the patients to general psychiatry if an underlying problem, such as ADHD, was detected but they considered that the specialist care acceptance of referrals was very low. This also applied to remittances to other specialist care units.

#### A wish for a mutual plan

The informants believed that their biggest challenges were the organizational structure. Lack of collaboration, teamwork and a mutual approach led to an ambiguity regarding the patient’s process through the care system.

*It would be better if there were routines, some template how to do, that it was not up to each and every one to decide how to proceed with the patients* (Participant 9).

Sitting down, discussing, and reconciling patient matters, having a mutual mindset, and a clear treatment plan, is believed to provide a better overview of the patient’s treatment. It could also improve the exchange of knowledge between different professions and provide a better basis for assessing the timing of different treatments. Meeting one another may prevent incongruity regarding the process.  For instance, if a physician changes the patient´s sick leave, affecting the psychologist’s treatment agenda and compromising the therapeutic treatment. Mutual plan is particularly important since many of the patients experience long duration of the illness, and this might cause difficulties in the relationship with the Swedish Social Insurance Agency. Thus, challenges around sick leave and lack of flexibility for return to work are stressors for both the patient and the therapist and in these situations coherent teamwork is important.

Some informants narrated how referral from other professions to psychologists is done to solve a problem as a quick fix, without considering the overall process for the patients.

This could lead to the patient lacking motivation or the psychologist struggling to identify a suitable focus for the treatment.

*You become some kind of anxiety reliever for everyone else at PHCC* (Participant 6).

The informants emphasized how the organizational barriers affect the possibility of offering equitable care. The PHCCs varying resources, different ideas about which treatment should be offered and varying interest from different professionals means that management and treatment of ED patients can vary considerably. According to most of the informants, lack of time is one of most important obstacles preventing good coordination for the patients.

#### The patient becomes the coordinator of their own care

Since most PHCCs lacked a mutual plan for coordinating the care, there was a risk that the patients are left with no one having an overview of their care process. Many informants believed that the decision regarding which care the patients are offered is dependent on which physician or psychologist they happen to meet.

*It is a big problem that many patients end up slipping between the cracks. Because no one has a holistic perspective. And the patients may not be that strong to keep in touch with everyone* (Participant nr 8).

This lack of overview regarding the holistic perspective forces many patients to become responsible for their own care process. Many informants also found it problematic that it was common for the patients to “wish for everything” regarding treatment in their quest to get well. However, the healthcare providers willingness to offer many different treatments could result in more stress for the patients instead of being helpful. Furthermore, the healthcare providers own perception that his/her efforts might not be sufficient often resulted in the patient being moved around “to see what works”, again with the risk of ambitious patients being overloaded.

*It falls on the patients that if they feel that it will be too much, they may well say no* (Participant nr 13).

An important point raised by the informants was that one reason for a patient requesting to participate in several different treatments was that sick-leave benefits are more easily granted if there is an ongoing treatment.

*They (the patients) can read between the lines that the Swedish Social Insurance Agency is a bit more benevolent if you say yes to different treatment options* (Participant nr 7).

Since internal referral of patients can be done between all healthcare providers within the PHCC and also to the rehabilitation centre, the rotation of patients usually happens without a coordinated long-term plan. This also means that different healthcare providers do not have the possibility of following up different treatments that have been offered. Hence, the patient´s continued path through care after psychological treatment is not known by the psychologist. This means poor control over whether the treatment has been sufficient and/or whether the patient’s newly acquired knowledge and strategies are being maintained.

## Discussion

This study explores psychologists´ experiences regarding the organisation of the care process and treatment of patients seeking care for stress-related exhaustion in primary care. The subcategories under psychologists’ assessment and treatment of ED included: deepening the medical diagnosis, addressing common symptoms, encountering unique needs in treatment, and finding the right timing can be difficult. The subcategories under organizational prerequisites included: management of patients with ED depends on available professional resources, lacking collaboration and communication, a wish for a mutual plan and the patient becoming the coordinator of his/her own care. An overall reflection of the results is that absence of a holistic perspective and lack of internal collaboration leads to poor utilization of the psychologist’s competence. Furthermore, the organisational structure of primary care is incongruent with the care process needed for these patients and does not support the idea of equitable care. This leads to a care process that sometimes could force the patients to be the carriers and coordinators of their own care.

### Fragmented care and lack of holistic overview of the care process

Lifestyle and behavioural issues play a major role for patients with stress-related mental health problems and a holistic overview is important for all professionals working with these patients. However, the informants describe that obtaining a holistic overview of the patient care process is seldom possible and they raised a concern about the random referral of these patients and a lack of clarity regarding how and/or when they or other professional category were included in the process. Perhaps, the most important issue to raise when discussing the fragmented care of patients with stress-related mental health problems is the view that traditional medical models will solve the root causes of stress-related mental health issues. Thus, in many cases the GPs are supposed to treat these patients without proper time, skills and training [10, 26], instead of incorporating collaborative models involving other professionals that can contribute with additional skills and training to judge and manage mental health problems [27, 28].

The informants described the internal referral as being governed by factors other than the patient’s care needs, including availability of different professions or the GP´s beliefs and preferences, something also shown in previous studies [10, 29]. Regarding availability of different professions, the informants raised the concerning issue of long waiting lists for a psychologist. Lack of staff resources could be one plausible reason explaining the long waiting lists. Another explanation for long waiting lists could be that methods from specialist psychiatric care, usually meaning long sessions and fixed end treatment times, are used without being adapted to a primary care setting, regarding generality and accessibility [30].

### Collaboration in primary care – a ceaseless challenge not yet solved

The results from this study show that collaborative care is poorly developed in primary care settings, at least in most of the settings included in this study. Primary care psychologists understand the importance of collaborative care, particularly for patients with a broad and complex diagnosis like ED which requires detailed characterization of what is specific for each individual case. However, the absence of organisational structure for collaboration and lack of time hampers the possibility of working in an interdisciplinary manner which leads to poor utilization of professional competences. For patients with ED, mutual plans and timing of interventions is of outmost importance, particularly since many of these patients present complicated psychological problems and are at risk of long-term illness. Thus, lack of collaborative care could in many cases hinder the patient’s possibility to recover.

One of the approaches applied to meet the challenges of more complex care has been to expand primary care settings with different professionals and roles, both by increasing the number of psychologists in primary care [31] and by introducing new roles including those of care manager and rehabilitation coordinator [20]. However, a collaborative structure cannot be achieved solely by adding more people or co-locating different professionals. Thus, the assumption that interdisciplinary health care teams will evolve naturally without training is considered erroneous empirical thinking [15, 32]. Different models have been developed, including the Primary Care Behavioral Health model that is being described as a prominent approach of integrating behavioural health services [33]. Thus, the model has been used in the US since the ‘90s [33] and an adapted form is being tested in Sweden. For the psychologist, the model means a changed role towards being more generalist which ultimately will result in increased availability of psychologists for patients in primary care settings.

#### Falling between the cracks in a non-supportive system

The patient perspective is raised by several informants. The informants describe a risk of the patients falling between the cracks in a non-supportive system, something that increases with a random passage through the healthcare system. Thus, the lack of collaborative care and structural processes puts the patient at a risk of being left alone and ending up as the carrier and coordinator of their own care. This is particularly problematic for patients suffering from impaired cognitive ability and lack of capacity to make decisions [34]. Lack of collaboration and coordination also increases the risk of both under- and over-dimensioned treatment which is described by the informants as a problematic consequence for the patients. Under- and over dimensioned treatment could also be a result of poor scientific evidence regarding treatment of patients with stress-related exhaustion [17, 18].

The patients also suffer from consequences of an inadequate referral process. Thus, “wrong” patients are being referred at the “wrong time”, resulting in time consuming and inadequate efforts for both the caregiver and patient. Furthermore, patients that are judged to need specialized psychiatry care can also fall between the crack since the informants describe having very little trust in the possibilities of referring these patients to the next level of care. Referral to psychiatry has been described by other researchers as a complicated and time-consuming process [10, 28, 35].

#### Moving forward

A changed disease panorama with more complex and long-term diseases, including mental health problems, places new demands on working multidisciplinary but this seems to be poorly addressed in today’s primary care services [11]. Different healthcare professional category, particularly psychologists, need to be involved early in the care process of patients with complex psychological needs, such as patients with ED. However, this study shows that the psychologist often become a referral instance for random patients, instead of being fully involved in the entire process for patients with complex psychological needs. This hampers the possibility for the psychologist to gain full control over the care process. The expert knowledge of different professions is poorly used, and GPs cannot be expected to bear the knowledge and skills of all professions in primary care. The dominance of the medical paradigm prevails even when caring for patients with complex psychological and social needs. Thus, new working methods including implementation of collaboration models are needed and this needs to be a conscious effort requiring both changes in organizational structures and changes in attitudes. The ultimate aim must be to build structures allowing all professions to use their professional skills in the best possible way, that best serves the patient’s needs.

Moving forward towards improved collaboration requires changes regarding policies and organizational structures, changed team processes and changed attitudes among different primary care professionals [16]. Furthermore, to achieve interprofessional collaboration, education and practice is needed and this will not occur by itself. An improved interprofessional collaboration would, from the psychologist’s perspective, mean better coordination and increased influence over the care process, follow-up, and evaluation of the patients. Collaborative practice would plausibly not only improve the patient’s situation but also the health care profession’s working conditions.

### Methodological considerations

To have a pre-understanding of the field explored in this study is considered a positive feature, but pre-understanding can also be an obstacle. Hence it is of great importance to be aware of one´s own preunderstanding and to be able to bridle it and by that ensure that it is the informants experiences that come to light. To follow a predetermined question guide that has a high degree of structuring can be considered limitation in a qualitative study, but in this case the questions functioned as a support to be compliant, and all questions could be rephrased and reordered to suit the different informants’ experiences. The question guide was also helpful in assuring that different dimensions of the informants’ experiences was covered. A relatively large number of interviews were conducted, and the results were also verified by the informants to ensure that the interpretation of the results were recognizable and in line with the original text material [23]. One limitation is that this study was conducted in a relatively small geographical area including professionals from relatively few primary care centers. Thus, we cannot draw firm conclusions that the situation described would be seen in all primary care centres in Sweden or in other countries. However, previous literature confirms that the challenges raised in this study are indeed described as general challenges within primary care and we have no reason to believe that the primary care centres included in this study would be considerably different from other centres. This study presents data from interviews with one profession and does not necessary reflect the perspective of other professions regarding the care process of patients with stress-related mental health problems. Other professions thoughts need to be illustrated to gain a comprehensive view of the situation.

Finally, it should be mentioned that in some cases the care was described as planned within the framework of a research project or education and in these cases the care is described as more clearly organized. These situations may thus not reflect the ordinary care process for these patients.

### Conclusion and clinical implications

The main conclusion of this study is that the care process of patients with stress-related mental health problems is perceived by psychologists in primary care to be ineffective and not congruent with the patient’s needs. The psychologists mostly have a positive view of their own work with the patients but the absence of a holistic overview of the care process, a lack of internal collaboration and poor utilization of the health care professionals’ competence leads to unstructured processes forcing the patients to be the carriers and coordinators of their own care. Furthermore, the situation does not support the idea of equitable care. The results from this study should be complemented by perspectives from other professionals as well as the patient’s perspective.

Knowledge from this study can hopefully be used to improve the care process of patients with stress-related mental health problems in primary care. It should, however, be pointed out that the hard-pressed primary care is faced with challenges in meeting suggestions such as those raised in this current study. Thus, it of highest priority to strengthen the primary care system with increased resources and better organizational framework if changes are to be seen.

### Electronic supplementary material

Below is the link to the electronic supplementary material.

Supplementary Material 1

## Data Availability

The datasets used and/or analysed during the current study are available from the corresponding author on reasonable request.
