# Supplementary material for: Psychologists’ involvement in and experiences of treating patients with stress-related exhaustion in primary care
Source: BMC Prim Care. 2024 Feb 12;25:56. doi: 10.1186/s12875-024-02287-7 (PMC10860213; doi:10.1186/s12875-024-02287-7)
Supplement: Supplementary file 1 — Supplementary Material 1 [file 12875_2024_2287_MOESM1_ESM.docx]

**Supplement 1**

**Interview Guide**

**Before the interview following steps were taken:**

• A suitable undisturbed place and time was found

• Introduction of the interviewer (where they are from, profession).

• An overview of the purpose of the interview was provided

• The overall purpose of the interview and the time frame (1-1.5 hours) was explained

• The importance of confidentiality was emphasized.

• The right to interrupt the interview/participation was informed

• Consent to recording, ensure the consent form is completed.

**Semi-structured interview guide:**

First, I would like you to describe your role at the healthcare center, your experience, and how long you have been working here.

At the end of the interview, there will be an opportunity for you to ask questions or discuss anything else you would like to mention.

I will ask a series of questions related to patients with diagnosed or suspected burnout syndrome. First, we will discuss the organization for this patient group, and then move on to diagnosis and treatment.

How do you organize the work for this patient group (with diagnosed or suspected burnout syndrome) at the healthcare center?

What is the process for these patients when they seek care at your center? (Who do they meet first?)

What treatment options are available for this patient category at the healthcare center? (Please provide specific examples. Do you differentiate based on severity?)

What criteria do you use to make decisions about different treatment options? (National guidelines, evidence, clinical experience, patient preferences, availability?)

Which other professionals do patients meet at the healthcare center, apart from you? (Psychologists, doctors, occupational therapists, social workers?)

-At what stage?

-How does the collaboration work?

-How do you receive feedback?

How are patients with this condition allocated to different providers?

Do patients receive continuous follow-up with you even if they are also seeing other providers?

What possibilities do you have as a provider to allocate the time you think is necessary?

How does your healthcare center approach individual assessment? (Or treatment according to routine?)

How do you consider the importance of collaboration with employers?

Which referral sources do you usually use? (Psychiatrists, specialized clinics, other doctors, vocational rehabilitation, physiotherapists?)

**Questions about the diagnosis of this patient group (confirmed or suspected):**

Who makes the diagnosis of burnout syndrome? (Is it not done at all? What are the diagnostic criteria?)

Is it common for the diagnosis to change during the course of treatment? How?

**Psychological treatment for patients with burnout syndrome (confirmed or suspected):**

At what stage of the illness do patients come to you?

If someone else determines whether the patient should see you or not, who is it, and how many of the total number of patients with this diagnosis do you estimate are referred to you?

How do you structure your treatment with the patient?

Do you follow a specific method, or does it vary?

What determines the method you use? (Patient, time, disease burden, where the patient is in the course of the illness?)

What do you consider the most important components of the treatment you provide?

How often do you meet with the patient? What are the intervals? When does it end?

Do you collaborate with any other professional during the patient's time with you?

How do you consider the timing of different treatment options?

What do you consider the biggest challenge with this patient group?

**Final question**

What do you think is missing from your education regarding burnout syndrome? (Diagnosis, treatment, other?)

Is there any question you were looking forward to that we haven't asked/anything else you would like to add?
